# Mapping the spatio-temporal structure of motor cortical LFP and spiking activities during reach-to-grasp movements

**DOI:** 10.3389/fncir.2013.00048

**Published:** 2013-03-27

**Authors:** Alexa Riehle, Sarah Wirtssohn, Sonja Grün, Thomas Brochier

**Affiliations:** ^1^Institut de Neurosciences de la Timone, UMR 7289, Centre National de la Recherche Scientifique - Aix-Marseille UniversitéMarseille, France; ^2^Riken Brain Science InstituteWako-Shi, Japan; ^3^Institute of Neuroscience and Medicine (INM-6), Computational and Systems Neuroscience, Research Center JülichJülich, Germany; ^4^Institute for Advanced Simulation (IAS-6), Theoretical Neuroscience, Research Center JülichJülich, Germany; ^5^Theoretical Systems Neurobiology, RWTH Aachen UniversityAachen, Germany

**Keywords:** cortical map, high-density recordings, monkey motor cortex, spiking activity, LFP

## Abstract

Grasping an object involves shaping the hand and fingers in relation to the object’s physical properties. Following object contact, it also requires a fine adjustment of grasp forces for secure manipulation. Earlier studies suggest that the control of hand shaping and grasp force involve partially segregated motor cortical networks. However, it is still unclear how information originating from these networks is processed and integrated. We addressed this issue by analyzing massively parallel signals from population measures (local field potentials, LFPs) and single neuron spiking activities recorded simultaneously during a delayed reach-to-grasp task, by using a 100-electrode array chronically implanted in monkey motor cortex. Motor cortical LFPs exhibit a large multi-component movement-related potential (MRP) around movement onset. Here, we show that the peak amplitude of each MRP component and its latency with respect to movement onset vary along the cortical surface covered by the array. Using a comparative mapping approach, we suggest that the spatio-temporal structure of the MRP reflects the complex physical properties of the reach-to-grasp movement. In addition, we explored how the spatio-temporal structure of the MRP relates to two other measures of neuronal activity: the temporal profile of single neuron spiking activity at each electrode site and the somatosensory receptive field properties of single neuron activities. We observe that the spatial representations of LFP and spiking activities overlap extensively and relate to the spatial distribution of proximal and distal representations of the upper limb. Altogether, these data show that, in motor cortex, a precise spatio-temporal pattern of activation is involved for the control of reach-to-grasp movements and provide some new insight about the functional organization of motor cortex during reaching and object manipulation.

## INTRODUCTION

The motor cortex is undoubtedly the first cortical area to be functionally examined in the history of neuroscience. In 1870, Fritsch and Hitzig did the first electrical stimulation experiments describing the topographical structure of motor cortex related to body segments (Fritsch and Hitzig, 1870). Almost 100 years later, Evarts (1964, 1966) started the first electrophysiological experiments in the awake behaving monkey to relate cortical activity to upper limb movements. Electrophysiological and anatomical studies have demonstrated the complex organization of body representation in the motor cortex of human and non-human primates. The motor effects evoked by intra-cortical micro-stimulation (ICMS) show systematic variations along the medio-lateral axis of the primary motor cortex (M1): ICMS at medial cortical sites in the precentral gyrus evokes lower limb movements, whereas ICMS at more lateral sites generate upper limb and head movements (Woolsey et al., 1952; Asanuma and Rosén, 1972; Kwan et al., 1978; Humphrey, 1986). Complementary studies revealed some additional variations in M1 and dorsal premotor (PMd) cortical organization along the antero-posterior axis. These observations suggest a clearly delineated somatotopic parcellation of motor cortical areas (Raos et al., 2003; Boudrias et al., 2010). However, converging evidence shows that body representation is not so strictly organized but characterized by a great degree of overlap between the cortical zones within M1 controlling nearby body parts (Park et al., 2001, 2004). This is particularly true within the distal upper limb representation in which there is little evidence of independent representation of the fingers (Schieber and Hibbert, 1993; Schieber, 2001).

On the functional side, it remains unclear how motor cortical organization as revealed by ICMS mapping relates to the activity of this cortical area during complex movements involving multiple body segments. Reach-to-grasp movements are particularly well suited to address this issue. These movements require the coordinated activation of arm and hand muscles to move the proximal and distal segments of the upper limb in a coherent way (Jeannerod, 1984; Jeannerod et al., 1995). Reaching requires activation of the arm muscles to transport the hand toward target objects, whereas grasping involves the activation of extrinsic and intrinsic hand muscles for hand preshaping and force control (Brochier et al., 2004; Stark et al., 2007). Following object contact, grasping also requires a fine adjustment of grasp forces for secure manipulation. Earlier studies suggest that the control of hand shaping and grasp force involves partially segregated motor cortical networks both during preparation and execution (Tokuno and Tanji, 1993; Rubino et al., 2006; Umilta et al., 2007; Vargas-Irwin et al., 2010; Bansal et al., 2012). However, it is still unclear how information originating from these networks is processed and integrated over motor cortical areas to give rise to a unified motor command.

One way to study the spatio-temporal modulations of neural activity during reach-to-grasp movements is to record simultaneously from an extended cortical territory through implanted microelectrode arrays. Vargas-Irwin et al. (2010) used 4 mm × 4 mm 100-electrode Utah arrays implanted in the upper limb representation just anterior to the central sulcus to analyze the spiking activity of single neurons during reach-to-grasp movements. They did not observe any systematic spatial dependence of neuronal firing with arm and hand movements, nor any related spatial partitioning of neuronal populations. However, Hatsopoulos and colleagues (Rubino et al., 2006; Hatsopoulos et al., 2011; Takahashi et al., 2011) used the same type of arrays to analyze the properties of local field potential (LFP) oscillatory activity in the beta frequency range (15–30 Hz) along the cortical surface close to the central sulcus. They described propagating waves of beta oscillations along the dominant axes of the motor cortex with respect to the proximal and distal motor representations in both humans and monkeys. These partially conflicting observations may be related to the observation that spiking activity and LFPs are not tightly correlated neuronal signals (Poulet and Petersen, 2008; Okun et al., 2010) and thus likely carry different information. LFPs can be recorded from the same electrode as single neurons and reflect mainly the spatially averaged synaptic input to neurons within a small volume around the electrode tip (Mitzdorf, 1985). It is plausible that the global LFP signal is more appropriate to capture gradual transitions within motor cortical maps than highly localized spiking activity.

Besides their oscillatory properties, LFPs are known to modulate in the time domain in relation to specific behaviorally relevant events. In motor cortical areas, LFPs exhibit a large multi-component movement-related potential (MRP) around movement onset (Donchin et al., 2001; Roux et al., 2006; Kilavik et al., 2010). However, little is known about the spatio-temporal distribution of MRPs across motor cortex in relation to task requirements. It is assumed that negative deflections of the LFP reflect excitatory inputs to the neurons in the local vicinity of the electrode tip and as such may promote an increase in spiking activity (Arieli et al., 1995; Destexhe et al., 1999). Following this assumption, one can hypothesize that the modulation of the amplitudes of the different MRP components may be characterized by specific spatio-temporal structures related to the motor cortical internal map.

In this paper, we use high-density intra-cortical recordings to study the temporal and spatial modulations of LFP and spiking activity during a delayed reach-to-grasp task. Neuronal activity was recorded by using a 100-electrode “Utah” array, chronically implanted in the precentral gyrus convexity. We first used the LFP signal to analyze the distinct MRP components and to explore how their peak amplitudes and latencies are spatially distributed over the cortical surface covered by the electrode array. Using this mapping approach, we showed for the first time that during movement execution, the spatio-temporal structure of the MRP reflects the complex physical properties of reach-to-grasp movement. In addition, we explored how the MRP structure relates to two other measures of neuronal activity: (i) the temporal profile of single neuron spiking activity at each electrode site and (ii) the somatosensory receptive field (RF) properties of single neuron activities. We observed that the spatial representations of LFP and spiking activities overlap extensively and relate to the spatial distribution of proximal and distal representations of the upper limb in motor cortex. Altogether, these data show that, in motor cortex, a precise spatio-temporal pattern of activation is involved for the control of reach-to-grasp movements and provide some new insight about the functional organization of motor cortex during reaching and object manipulation. Preliminary data were presented in Brochier and Riehle (2011) and Riehle and Brochier (2012).

## MATERIALS AND METHODS

### BEHAVIORAL TASK

One adult female macaque monkey (*Macaca mulatta*), weighing 4.5 kg, was used in the experiment. All animal procedures were approved by the local ethical committee (authorization A1/10/12) and conformed to the European and French government regulations.

**FIGURE 1:**
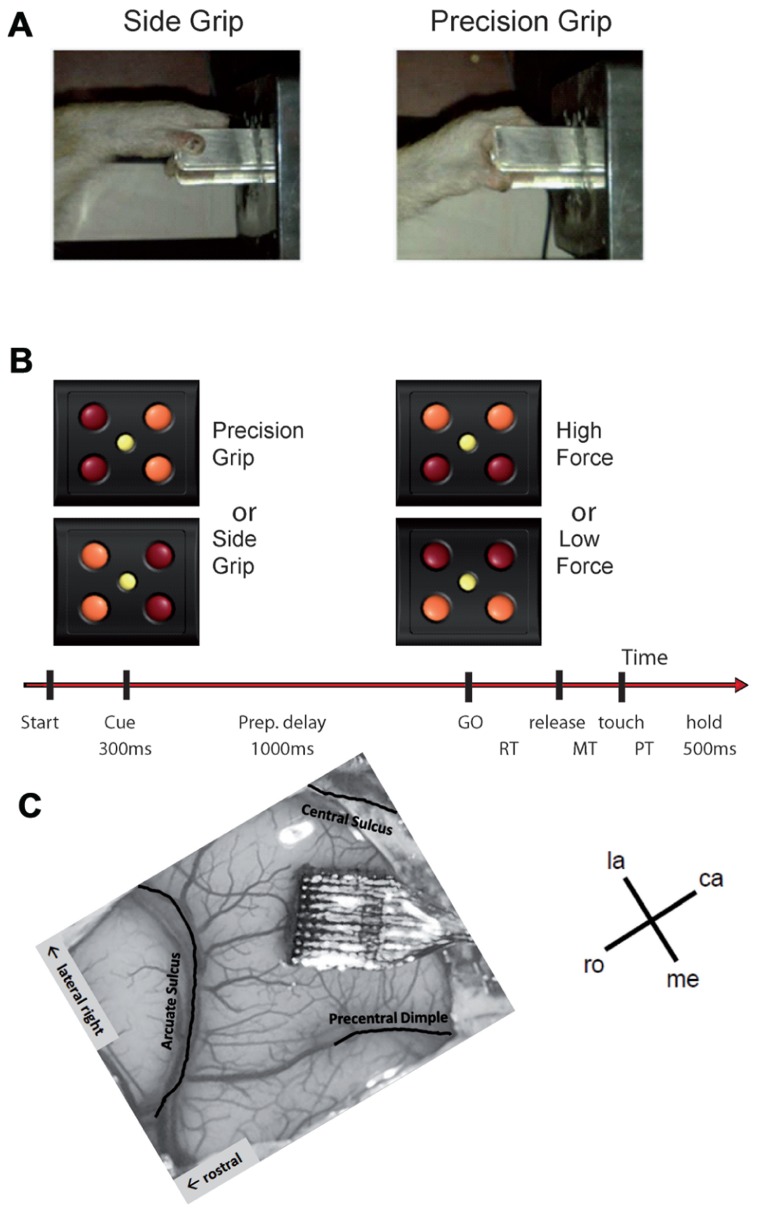
**(A,B)** Experimental design (see text for details). **(C)** Implanted Utah array in the motor cortex of our monkey. The picture is rotated such that the array is oriented in the same way as the maps shown in **Figures 4, 8**, and **9**. la, lateral; me, medial; ro, rostral; ca, caudal.

The monkey was trained to perform an instructed delay reach-to-grasp task to obtain a food reward (apple sauce), using the left hand, and sat in a custom-made primate chair in front of the experimental apparatus with the non-working arm loosely restrained in a semi-flexed position. The unrestrained working hand rested on a switch positioned at waist-level, 5 cm lateral to the midline. The target object was a stainless steel parallelepiped (40 mm × 16 mm × 10 mm) attached to the anterior end of a low-friction horizontal shuttle and rotated at a 45° angle from the vertical axis (see **Figure 1A**). It was located 13 cm away from the switch at 14 cm height. The object had to be grasped and pulled with the working hand using one of two different grips: a precision grip (PG) by placing the tips of the index and the thumb in a groove on the upper and lower sides of the object, respectively, or a side grip (SG), by placing the tip of the thumb and the lateral surface of the index on the right and left sides, respectively (**Figure 1A**). The object weight could be set to one of two different values (100 or 200 g) by means of an electromagnet inside the apparatus. Thus, the force required to pull the object was either low force (LF) or high force (HF) when the magnet was turned off or on, respectively. Changes in object weight occurred between trials and were undetectable by the monkey. The apparatus provided a continuous measure of the grip and pulling load forces by means of force sensitive resistances (FSR). In addition, a hall-effect sensor measured the horizontal displacement of the object over a maximal distance of 15 mm.

A square of four red light-emitting diodes (LEDs) with one yellow LED in its center was used to display the instruction cues (**Figure 1B**). The LEDs were inserted in the apparatus just above the target object. Illumination of the two left or right red LEDs instructed the monkey to perform a SG or a PG, respectively. Illumination of the two bottom or top LEDs instructed the monkey that pulling the object required a LF or HF, respectively.

The task was programed and controlled using LabVIEW (National Instruments Corporation, Austin, TX, USA). The trial sequence was as follows (see **Figure 1B**). The monkey had to close the switch with the hand to initiate a trial. After 400 ms, the central yellow LED was illuminated for another 400 ms, followed by the preparatory cue, illuminated for 300 ms, which instructed the monkey about the grip (PG or SG) required to perform the trial. Cue extinction was followed by a 1-s preparatory delay. At the end of this delay, the GO signal provided the remaining information about the force and also served as imperative signal instructing the monkey to release the switch to reach and grasp the object. Following object grasp, the monkey had to pull the object into a narrow position window (4–14 mm) and to hold it there for 500 ms to obtain the reward. In case of grip error, the trial was aborted and all four LEDs were flashed as a negative feed-back. The reaction time (RT) was defined as the time between the GO signal and switch release and the movement time (MT) the time between switch release and grip force onset as detected by the FSR by using a fixed threshold. The monkey was required to keep RT and MT below 700 ms to be rewarded. Five to 10 recording subsessions of about 10–15 min each were recorded per recording session, one session per day, up to five sessions per week. During each subsession, the four trial types, i.e., a combination of SG–LF, SG–HF, PG–LF, and PG–HF, were presented at random with equal probability. The monkey usually achieved a total of 100–200 successful trials/subsession.

### SURGERY

When the monkey was fully trained in the task and obtained 85% of correct trials, a 100-electrode Utah array (Blackrock Microsystems, Salt Lake City, UT, USA) was surgically implanted in the motor cortex contralateral to the working hand. The array had an arrangement of 10 × 10 iridium oxide electrodes, each of them 1.5 mm long, with an inter-electrode distance of 400 μm. The surgery was performed under deep general anesthesia using full aseptic procedures. Anesthesia was induced with 10 mg/kg i.m. ketamine and maintained with 2–2.5% isoflurane in 40:60 O_2_–air. To prevent cortical swelling, 2 ml/kg of mannitol i.v. was slowly injected over a period of 10 min. A 20 mm × 20 mm craniotomy was performed over the motor cortex and the dura was incised and reflected. The array was positioned on the cortical surface 3 mm anterior to the central sulcus at the level of the spur of the arcuate sulcus (**Figure 1C**). The array was inserted using a pneumatic inserter (Array Inserter, Blackrock Microsystems) and covered with a sheet of an artificial non-absorbable dura (Gore-tex). The real dura was sutured back and covered with a piece of an artificial absorbable dura (Seamdura, Codman). The bone flap was put back at its original position and attached to the skull by means of a 4 mm × 40 mm strip of titanium (Bioplate, Codman). The array connector was fixed to the skull on the opposite side with titanium bone screws (Bioplate, Codman). The skin was sutured back over the bone flap and around the connector. The monkey received a full course of antibiotics and analgesic before returning to the home cage.

### RECORDINGS

Data were recorded using the 128-channel Cerebus acquisition system (Blackrock Microsystems, Salt Lake City, UT, USA). The signal from each active electrode (96 out of the 100 electrodes were connected) was pre-processed by a head stage with unity gain and then amplified with a gain of 5000. The signal was filtered in two different frequency bands to split into LFPs (0.3–250 Hz) and spiking activity (0.5–7.5 kHz). The LFPs were sampled at 1 kHz and saved on disk. On every channel, the experimenter set a threshold online for spike selection. All waveforms crossing the threshold were sampled at 30 kHz and snippets of 1.6 ms duration were saved for offline spike sorting. All behavioral data such as stimuli, switch release, force traces for thumb and index fingers, and object displacement were fed into the Cerebus, sampled at 1 kHz and stored for offline analysis. During the offline spike sorting (Offline Spike Sorter, version 3, Plexon Inc., Dallas, TX, USA), spike clusters which were separated significantly from each other and with less than 1% of inter-spike intervals (ISIs) of 2 ms and less were considered as single units (single-unit activity, SUA), whereas less well separated clusters and/or more than 1% of 2 ms ISIs were considered as multi-unit (multi-unit activity, MUA) recordings.

### DATA SELECTION

Data were obtained from 57 recording sessions over a period of more than 7 months. For LFP analysis, we selected 18 recording subsessions from different sessions. Three criteria guided the selection. Each selected subsession had to contain a sufficient number of trials (at least 100), it should show as few artifacts as possible and the selected subsessions should homogeneously span the entire 7 months of recording. For spike data, 11 recording subsessions were selected using the same criteria. These subsessions were different from the LFP subsessions since they were also selected to get as many (at least 80) recorded single neurons as possible per subsession.

### DATA ANALYSIS

All data were analyzed using Matlab (The MatWorks Inc., Natick, MA, USA).

The timing of the behavioral events in the different tasks was calculated offline. The object touch was calculated from the first derivative of the grip force measured at the thumb (i.e., the grip force rate, GFR). In each trial, it corresponded to the time after switch release at which the GFR passed a threshold arbitrarily set to max_GFR_/25 (where max_GFR_ corresponds to the peak of GFR in the current trial). The object pull was computed from the object displacement measure and corresponded to the time at which the object entered the position window. The time difference between switch release, object touch, object pull, and reward corresponded to the RT, MT, and hold time (Hold), respectively.

**FIGURE 2:**
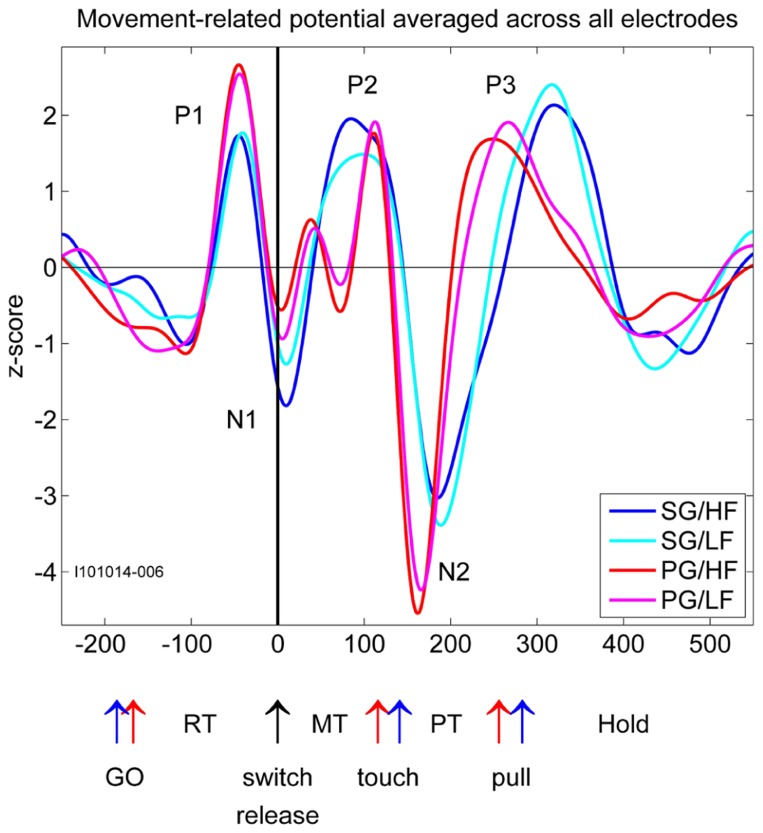
**Movement-related potentials (MRPs) of the LFP for each of the four trial types averaged across all electrodes recorded in one selected subsession.** Time in millisecond around switch release (t0). SG, side grip; PG, precision grip; LF, low force; HF, high force; RT, reaction time; MT, movement time; PT, pulling time; GO, GO signal occurrence; touch, touching the object; pull, arriving in the hold window. The red and blue arrows correspond to the timing of the trial averaged events during PG and SG trials, respectively.

**FIGURE 3:**
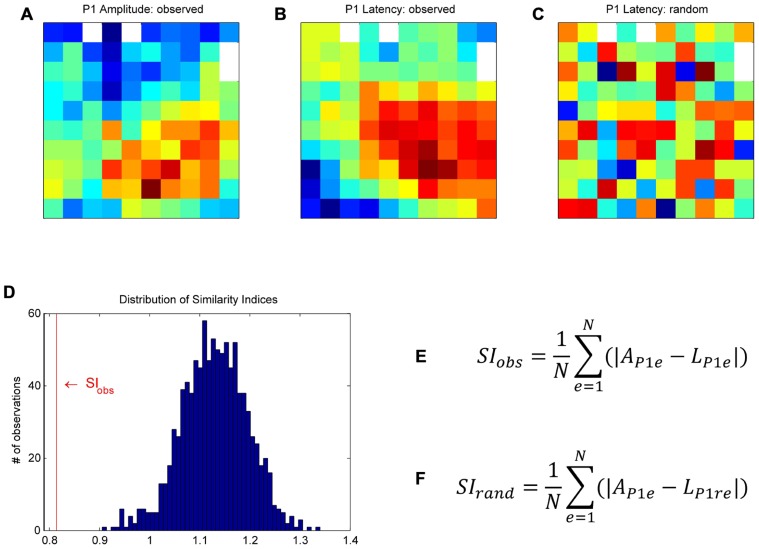
**Bootstrap procedure to assess whether the similarity between a given pair of maps could have occurred by chance (for more details, see Materials and Methods)**. As an example, we used the maps of the P1 peak amplitudes **(A)** and their latencies **(B)**. First, in order to homogenize the data to be compared between maps, we *z*-scored the data on each map individually. We then calculated a similarity index (SI) between the two maps of observations. At each electrode, we computed the absolute difference between the P1 amplitude and the P1 latency, and averaged these values across all electrodes to obtain the similarity index, SI_obs_ **(E)**, where *A*_P1e_ is the peak amplitude of the P1 component at electrode e, *L*_P1e_ the peak latency of the P1 at the same electrode, and *N* the number of electrodes. For the bootstrap, the P1 latency values of the original map were randomly shuffled in space to create a “random P1 latency map” **(C)**. We calculated the SI between this “random latency map” and the original P1 amplitude map, SI_rand_ **(F)**. This procedure was repeated 1000 times to build a distribution of SI_rand_ **(D)**. SI_obs_ was compared to the distribution of SI_rand_ to assess if the similarity between the two observed maps was significant.

Local field potential signals from each electrode and from each recording subsession were processed independently. For some subsessions, visual inspection of the data showed that in very few electrodes, the signal was corrupted by recurrent artifacts in almost all trials. These electrodes were excluded from the analysis. For each remaining electrode, the raw LFP signal was band-pass filtered in the range of 3–15 Hz (fourth order Butterworth filter). This frequency range corresponds to the main frequency band of the MRPs (Kilavik et al., 2010) excluding both the prominent beta oscillations (20–30 Hz; Kilavik et al., 2012a) and slow frequency modulations (<2 Hz) such as the contingent negative variation (CNV) occurring during an instructed delay (Walter et al., 1964; Zaepffel and Brochier, 2012). In each trial, the LFP was aligned to switch release and cut in a time window starting 500 ms before and ending 1000 ms after switch release, and then *z*-scored across all trials. At every time point in this window, the mean and standard deviation of the LFP signal was computed across all trials of each trial type. We discarded each individual trial in which the signal exceeded the mean LFP ±2 standard deviations at any time point. This procedure was used to reject outlier trials that may be corrupted by non-physiological artifacts. Mean LFPs on each electrode exhibited a large MRP with three positive components alternating with two negative components (**Figure 2**, only -250 to 550 ms with respect to switch release are shown here). We labeled these components P1, N1, P2, N2, and P3. In some sessions, the P2 component for PG trials was divided in two distinct subcomponents. We labeled these two subcomponents P2-1 and P2-2. Each component of each trial type was analyzed separately. We first determined its absolute peak amplitude and its peak latency in relation to switch release for each electrode in each subsession in appropriate time windows covering the component. We then analyzed how the peak amplitude and latency across all subsessions varied spatially between electrodes. To do so, we used a 10 × 10 matrix to represent the cortical surface covered by the 100-electrode array. In all figures, we oriented this matrix so that the top row is parallel to the central sulcus (see **Figure 1C**), and the left upper corner represents the lateral electrodes, the left lower corner of the matrix being closer to the arcuate sulcus (toward PM), representing the rostral electrodes. The right upper and lower corners of the matrix represent the caudal and medial electrodes, respectively. We used a color code to represent the peak amplitudes of the component at each electrode location on the matrix. In **Figure 3A**, an example of peak amplitude maps can be seen, computed from SG–HF trials, in which the largest and smallest amplitudes were represented in red and blue, respectively. To represent the spatial modulation of the peak latencies (see **Figure 3B**) we used another matrix for display. On the peak latency map, earliest and latest peaks with respect to switch release were represented in red and blue, respectively. We repeated the same procedure to create an amplitude and latency map for each of the five MRP components and for each of the four trial types across all subsessions.

In each recording subsession, these maps showed that the five MRP components varied in amplitude and latency across the array. In a first analysis, we tested for each MRP component if the layout of these maps was consistent across recording subsessions. For this purpose, we compared the MRP map in each subsession to the map in all the other subsessions, one by one. For each pair of subsessions, we computed the pairwise correlation coefficient between the amplitudes of the component on each electrode in the two subsessions. We then counted the number of significant correlations (*p*<0.05) across all possible pairs of subsessions. The same method was applied to quantify the consistency of the peak latency maps of each MRP component. The consistency across subsessions of the amplitude/latency map of a given component was considered to be significant if more than 95% of the subsession pairs showed a significant correlation (see black fields in **Figure 5**).

Since the maps were highly consistent across subsessions (see Consistency of Maps Across Sessions and **Figure 5**), the following analyses were done on peak amplitude and latency maps averaged across all 18 subsessions. We raised three different issues: Were the peak amplitude maps different for the four trial types? Did the spatial modulation of MRP peak amplitude relate to the spatial modulation of its latency? Was there any similarity between the amplitude maps of the five MRP components? To address these issues, we used a bootstrap procedure to assess whether the similarity between a given pair of maps could have occurred by chance. This procedure is described below by using, as an example, the comparison between the P1 amplitude and the P1 latency maps (see **Figure 3**). First, in order to compare two maps, we had to normalize the two data sets to obtain a similar scale. To do so, we *z*-scored the P1 peak amplitude at each electrode by the mean and standard deviation of the P1 peak amplitudes computed across all electrodes. The same normalization was applied to the P1 peak latency map. We then calculated a similarity index (SI) between the two maps of observations. At each electrode, we computed the absolute difference between the P1 amplitude and the P1 latency, both normalized and in arbitrary units. These values were then averaged across all electrodes to obtain the SI (SI_obs_).

$${\text{SI}}_{\text{obs}}=\frac{1}{N}\overset{N}{\underset{\text{e}=1}{\Sigma }}\left|A_{\text{ple}}-L_{\text{ple}}\right|.$$

Where *A*_P1e_ is the peak amplitude of the P1 component at electrode e, *L*_P__1e_ the peak latency of the P1 at the same electrode, and *N* the number of electrodes. For the bootstrap, the normalized P1 latency values of the original map were randomly shuffled in space to create a “random P1 latency map” (see **Figure 3C** for an example map). We calculated the SI between this “random latency map” and the original P1 amplitude map (SI_rand_). This procedure was repeated 1000 times to build a distribution of SI_rand_ (see **Figure 3D**). The 25th and 975th SI_rand_ defined the upper and lower limits of the confidence interval. SI_obs_ was compared to the distribution of SI_rand_ to assess if the similarity between the two observed maps was significant. If SI_obs_ is at the lower tail of the distribution, as in **Figure 3D**, the two maps match positively, that is they significantly cover the same/similar space on the matrices. If SI_obs_ is at the upper tail of the distribution, the two maps match inversely, that is when a value is high on one map, it is rather low on the other map. The same bootstrap procedure was used to compare the peak amplitude and latency maps for the five MRP components, the peak amplitude and latency maps of each component to the peak amplitude and latency maps of the other components and the peak amplitude maps of the different trial types.

## RESULTS

### LOCAL FIELD POTENTIALS

#### Maps of peak amplitudes and latencies on the cortical surface covered by the array

**FIGURE 4:**
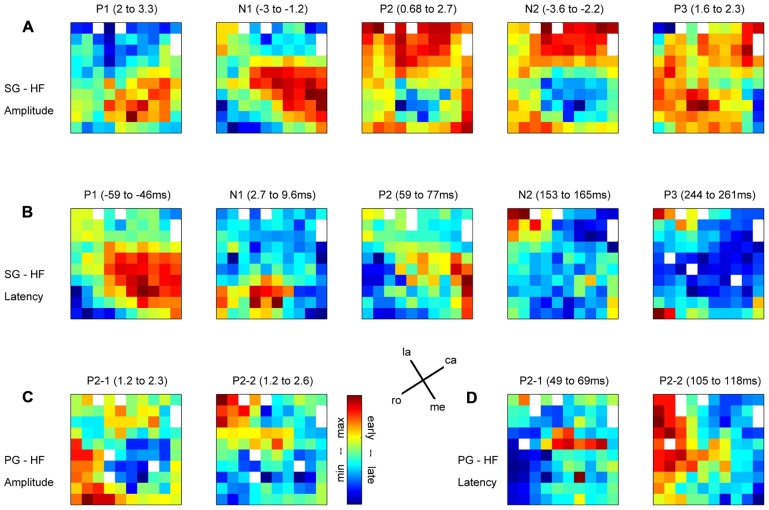
**Maps of peak amplitudes (A,C) and latencies (B,D) of the different components of the MRP averaged across all selected subsessions; *n* = 18 in (A,B); *n* = 12 in (C,D).** Color code is in absolute values and adapted to each component separately. Above each map, the MRP component and the min–max values are indicated in *z*-score for the amplitudes **(A,C)** and in millisecond with respect to switch release for the latencies **(B,D)**. **(A,B)** Side grip–high force; **(C,D)** precision grip–high force. White squares are either the four inactive electrodes (see their position in A) or eliminated electrodes because of outliers. la, lateral; me, medial; ro, rostral; ca, caudal; see **Figure 1C**.

In relation to our complex reach-to-grasp task, the MRP of the LFP contains five distinct components around movement onset (switch release). **Figure 2** shows an example of MRPs averaged across all electrodes of the array recorded during one recording subsession for each of the four trial types. The five components occur at specific time points during reaching and grasping: P1 occurs between the GO onset and switch release, N1 around switch release, P2 between switch release and object touch, N2 during object pulling, and finally P3 during the object hold. Although this temporal sequence could suggest that each component is linked to a specific task event, we observed that the average MRPs of all five components are the largest when single trial LFPs are aligned to switch release. Therefore, all our analyses included here were done using LFPs aligned to switch release. **Figure 4** shows the peak amplitude (**Figure 4A**) and peak latency (**Figure 4B**) maps for each of the five components during SG–HF trials, averaged across the 18 selected subsessions of recording (see Materials and Methods for details). **Figures 4C,D** show the amplitude and latency maps, respectively, for the two P2 subcomponents in PG–HF trials. The spatial layout of these maps shows clear differences between the individual components. The highest amplitudes and the earliest peak latencies of P1 and N1 are localized mainly in the lower part of the maps, representing the rostral electrode positions closer to the precentral dimple (see **Figure 1C**). In contrast, the later components, P2 and N2, are largest in the upper part of the map, representing the electrode positions closer to the central sulcus. P3 has no clear localization. The two P2 subcomponents could clearly be separated in 12 out of the 18 subsessions and were analyzed separately as shown in **Figures 4C,D**. Interestingly, striking differences are observed between the map layouts of the two subcomponents. The amplitude of the first subcomponent is largest at the bottom of the map whereas the amplitude of the second subcomponent is largest at the top. The latency maps also show that for these two subcomponents, the peak occurs earlier on specific electrodes at the center and on the left border of the map for P2-1 and P2-2, respectively. In the following section, we questioned if the spatial organization of these average maps results from a systematic spatial distribution of the five LFP components in individual recording subsessions.

#### Consistency of maps across sessions

**FIGURE 5:**
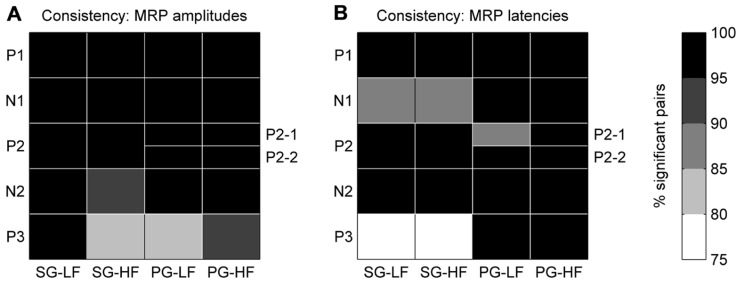
**Consistency of peak amplitude **(A)** and peak latency **(B)** maps across all selected subsessions** The gray color code corresponds to the percentage of significant data pairs for each component and behavioral condition. The black fields indicate that more than 95% (*p*<0.05) of the pairs were statistically significant. Note, for PG the P2 component is split in two subcomponents, P2-1 and P2-2.

In order to test the consistency of the peak amplitude and latency maps from subsession to subsession, we calculated the correlation between the maps for each possible pair taken from the 18 subsessions (*n* = 153). The correlation between maps of the P2-1 and P2-2 subcomponents in PG trials could be calculated only in 12 subsessions (*n* = 66; see Maps of Peak Amplitudes and Latencies on the Cortical Surface Covered by the Array). **Figure 5A** shows that for all components but P3, the between subsession correlation of the amplitude maps is significant in more than 95% of the pairs (black fields). Also, for all components but the N1 and P3 during SG (see **Figure 5B**) the between subsession correlation of the latency maps was significant in more than 95% of the pairs. The lowest amount of significant pairs was found for the P3 latencies in SG, being nevertheless higher than 75%. In other words, the maps of both peak amplitudes and latencies were highly consistent over the 7 months of recording and, thus, we performed the following analyses on the data sets averaged across all recording subsessions.

#### Spatial representation of behavioral conditions

In order to test if the different behavioral parameters such as grip and force are differently represented on the motor cortical surface covered by the array, we used a bootstrap procedure with 1000 iterations (see Materials and Methods, **Figure 3**). This procedure quantifies the likelihood that the similarity between maps could have occurred by chance. Here we compared the peak amplitude maps of each component between trial types. For the P2 component in PG we chose the larger subcomponent, i.e., the P2-2. This comparison reveals that the maps for both the two grip types and the two force types are almost identical (*p*<0.001). For that reason, we selected only HF trial types for further analyses. However, since the shapes of the MRPs related to SG and PG strongly varied (see **Figure 2**), we analyzed SG and PG separately.

#### Spatial relation between peak amplitude and latency

**FIGURE 6:**
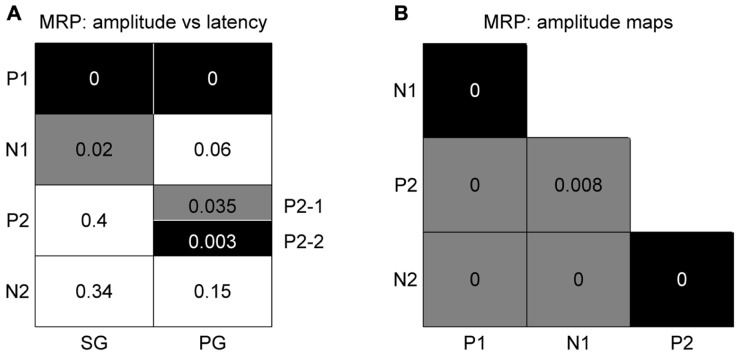
**Significance levels of comparisons between maps of MRP components for high force trial types only**. Black: statistically significant (*p*<0.05) positive match; gray: statistically significant negative match; white: not significant. The numbers correspond to the *p*-value for each combination. **(A)** Peak amplitude vs peak latency maps. **(B)** Comparison between peak amplitude maps of different components. The results for SG and PG are identical.

In order to determine if there is any spatial relationship between the peak amplitude and its latency for individual MRP components, we used the same bootstrap procedure as described in Section “Spatial Representation of Behavioral Conditions” with 1000 iterations (see Materials and Methods). The main result is that there is a significant (*p*<0.05) match between amplitude and latency for the P1 component obtained for the two grip types (black squares in **Figure 6A**), meaning that the higher the P1 amplitude, the earlier it occurs. Less systematic effects are observed for the other components. The N1 component shows a significant inversed match between the amplitude and latency maps, but only for SG trials (gray square in **Figure 6A**). This inversed match indicates that the higher the amplitude of the N1 peak, the later it occurs. The two subcomponents of the P2 in PG trials show opposite effects. The P2-2 amplitude map matches the latency map, whereas P2-1 amplitude and latency maps show a inversed match. The spatial organization of the P3 component is very poor (see **Figure 4**) and thus it is not further considered for comparison.

#### Different spatial representations of the individual components

We again used the same bootstrap procedure as described above to test the similarity between the amplitude maps of the different MRP components (see **Figure 6B**). A black square indicates that the maps are significantly more similar than predicted by chance, i.e., that the maps significantly match. A gray square indicates the reverse, i.e., that the maps show a significant reversed layout. This comparison shows that all combinations were statistically significant, where the two early components (P1–N1) and the two late components (P2–N2) share the same spatial representations. On the other hand, the maps of P1–P2, P1–N2, N1–N2, and N1–P2 have an opposite representation on the cortical surface. The same result was obtained for both SG and PG. As in the previous comparison, the poor spatial organization of the P3 component precludes it from further comparison.

### MAPPING THE SINGLE NEURON SPIKING ACTIVITY ACROSS THE CORTICAL SURFACE

**FIGURE 7:**
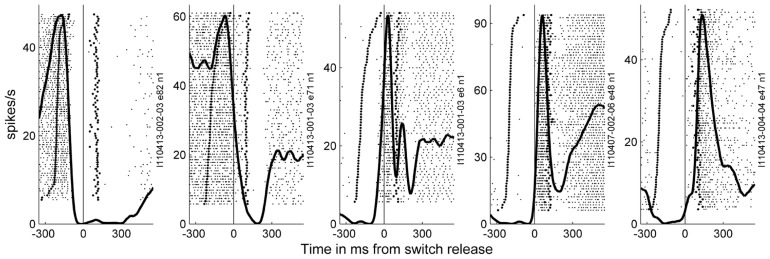
**Examples of the activity of neurons recorded during a few of the selected subsessions**. Spiking data were aligned to switch release (t0) as the MRPs of the LFP. In the raster displays, each horizontal line corresponds to a trial, and each small dot to the occurrence of a spike. Trials were aligned according to increasing reaction times. In each example, the data recorded during 59–82 trials during SG–HF are shown. The first raw of large dots corresponds to the occurrence of the GO signal in each trial, and the large dots in the second raw correspond to object touch. Spiking data were averaged across all trials and represented as post-stimulus time histogram (PSTH, thick line). It can clearly be seen, that each neuron exhibits only one peak of activity, but peaks at a different moment in time around switch release.

**FIGURE 8:**
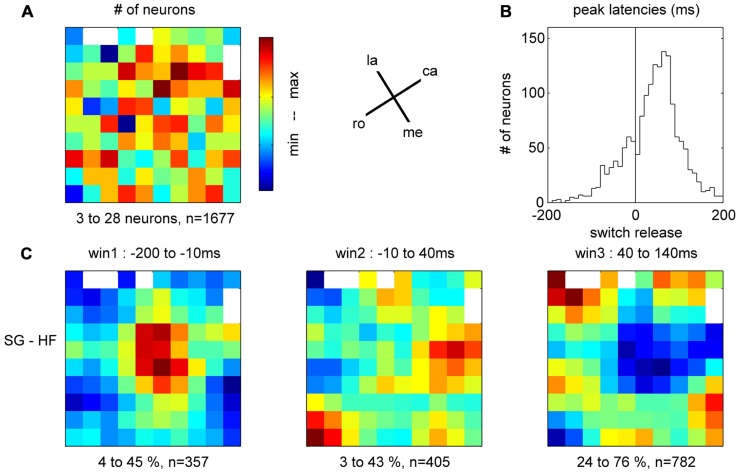
**(A)** Total number of neurons recorded on each electrode during the 11 selected subsessions, peaking during ±500 ms around switch release. la, lateral; me, medial; ro, rostral; ca, caudal; see **Figure 1C**. **(B)** Peak latencies of all neurons with respect to switch release (t0). **(C)** Amount of neurons peaking during one of the three time windows corresponding to the first three components of the MRP of the LFP, expressed as percentage of neurons from all neurons peaking around switch release (±500 ms, *n* = 1677) on each electrode. For the color code, the min (blue)–max (red) values of the percentages are indicated below each map, as well as the total number of neurons peaking during the respective window.

In the 11 subsessions of data selected for the analysis of the spike data (see Materials and Methods), single neuron activities were recorded from almost all electrodes, leading to 83–119 well-sorted single neurons (SUA) and 27–90 MUAs. Across these 11 recording subsessions, a total of 1058 SUAs and 809 MUAs were discriminated. The similarity of the SUAs across sessions was not systematically assessed. However, visual inspection of the spike waveforms, inter-spike-interval histograms and post-stimulus time histograms (PSTHs) in the task suggested that most of the SUAs isolated in different recording subsessions actually corresponded to different neurons. Therefore for the purpose of this study, all neurons in all subsessions were considered as independent neurons and included in the analysis. **Figure 7** shows five examples of the activity of single neurons recorded during some of the selected subsessions. Spiking data were aligned to switch release, as were the MRPs of the LFP. It can clearly be seen, that each neuron exhibits only one peak of activity, but peaks at a different moment in time around switch release. **Figure 8A** shows the numbers of neurons (SUAs and MUAs) discriminated on each electrode across the 11 subsessions which had a peak in their firing rate during a window of ±500 ms around switch release (*n* = 1677 out of the 1867 recorded neurons). These numbers appear evenly distributed across the array. Here we investigated the relationship between spiking activity and MRP modulations (**Figure 4**). More specifically, we questioned if the peak activity of single neurons could be related in space and time to the peaks of the different MRP components. We restricted this comparative analysis to the first three MRP components, P1, N1, and P2, which temporally best related to the peak occurrences of the spiking activity (**Figure 8B**). After aligning spike data in each trial to switch release (t0), we computed the mean firing rate across all trials (PSTH) with a temporal resolution of 1 ms, which was smoothed with a Gaussian filter (length 50 ms) and converted to spikes per second. We then determined for each neuron the latency of the peak firing rate with respect to switch release. **Figure 8B** presents the distribution of the peak latencies for the 1673 neurons (SUA and MUA). This distribution shows that although the peak activity of most neurons occurs after t0, an important proportion of neurons do actually peak before t0. We analyzed if the proportion of neurons peaking before and after t0 is equally distributed in space across the array. In relation to the MRPs, we selected three discrete time windows around the peak latency of the three MRP components, win1 from -200 to -10 ms (P1), win2 from -10 to 40 ms (N1), and win3 from 40 to 140 ms (P2). We then calculated the percentage of neurons recorded at a given electrode peaking during a given time windows with respect to the total number of all recorded neurons peaking in relation to switch release (*n* = 1677). The result of this analysis is presented in **Figure 8C** for the three time windows. The data were smoothed over the array by averaging the values obtained on each electrode with those obtained on all directly adjacent electrodes and color coded. It can be seen that the neurons peaking during the first time window (win1) are mostly located in the center right part of the array. A reverse pattern is observed in the third time window (win3) during which most of the neurons that are peaking were recorded on the array borders.

### SOMATOSENSORY PROPERTIES OF SPIKING ACTIVITY

**FIGURE 9:**
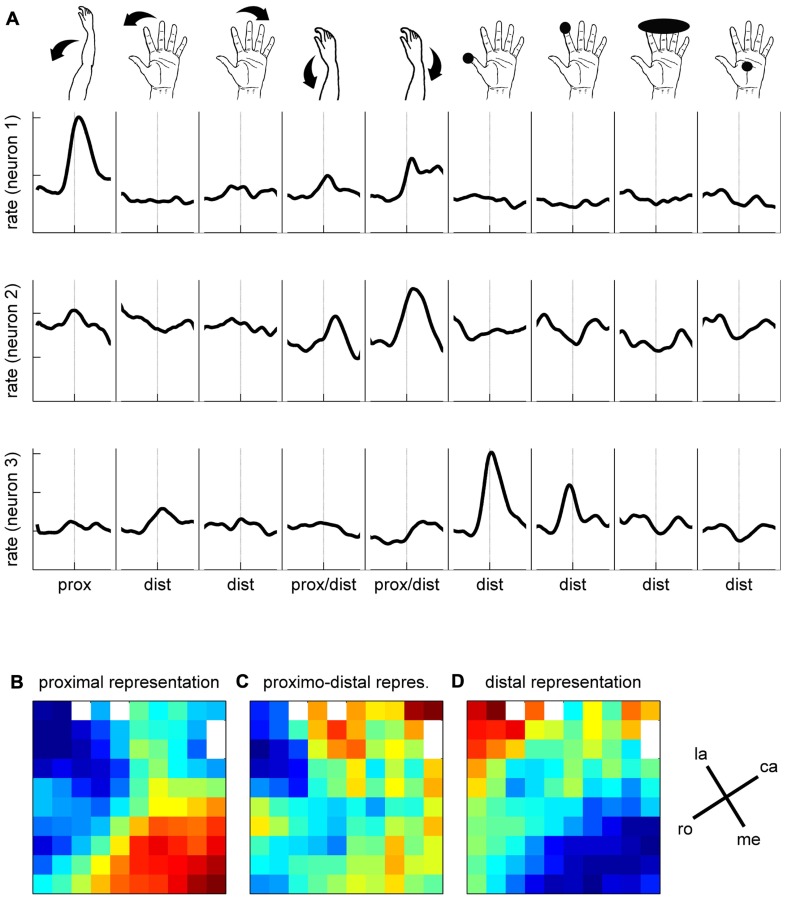
**Somatosensory properties of spiking activity**. **(A)** Responses of three simultaneously recorded neurons to nine stimulus conditions, as indicated by the drawings above. Mean firing rates are indicated in *z*-score, averaged across ~30 trials. For each stimulation condition, the mean firing rate is presented ±400 ms around the trigger signal (dashed lines). **(B–D)** Maps of somatosensory properties. Color code indicates min (blue) to max (red) activation, averaged across all neurons recorded during 3 days on each electrode of the array. White squares correspond to the inactive electrodes of the array. la, lateral; me, medial; ro, rostral; ca, caudal; see **Figure 1C**.

To better characterize the functional properties of the cortical zone covered by the array, we explored the somatosensory RFs of all the recorded neurons in three sessions over three consecutive days. To do so, we applied passive movements or tactile stimulations on different parts of the left upper limb (i.e., the limb used during the task) while recording the spiking activity on all electrodes simultaneously. Each particular stimulation was tested separately. It was applied about 30 times at 0.3 Hz and synchronized with a trigger signal generated by the experimenter for offline analysis of the evoked responses. The trigger consisted of a switch operated by the experimenter’s foot when applying the stimulus. We grouped the stimulations into three categories with respect to their location on the upper limb (see inset in **Figure 9A**). *Distal* stimulations were applied on different parts of the hand and fingers and included light touch of the thumb tip, of the inner side of thumb, of the index tip; of digits 2–5 tips, of the hand palm; passive thumb adduction, abduction, or flexion; passive index abduction or extension, passive digits 2–5 flexion or extension; simultaneous flexion of the thumb and index in a passive PG. *Proximo-distal* stimulations were applied to the wrist and included passive wrist flexion, pronation or ulnar deviation. *Proximal* stimulations were applied to the elbow or shoulder and included passive elbow flexion or extension and passive shoulder elevation or lowering. In each of the three recording sessions, we tested up to nine different stimulations using at least one stimulation of each category (distal, proximo-distal, and proximal). In the three sessions in which we tested the RFs, we recorded 90, 91, and 103 SUAs and 78, 79, and 69 MUAs, respectively. For each neuron (SUA and MUA), we computed a PSTH for each stimulation type separately. **Figure 9A** illustrates the responses of three simultaneously recorded neurons to the nine stimulation types used in the first recording session. The spiking activity evoked by each stimulus was analyzed in a ±400 ms window around the experimenter’s trigger (dashed lines). The mean spike count was computed with a temporal resolution of 1 ms across all trials (*N* ~ 30), smoothed with a Gaussian filter (length 50 ms) and converted to spikes per second. Each PSTH was then *z*-scored by the mean and standard deviation of the firing rate across all stimulation types. By doing so, the relative amplitude of the responses could be directly compared between neurons. Neuron 1 responds strongly to the elbow flexion, moderately to the wrist stimulation and very weakly to the distal stimulation. In contrast, the response of the second neuron is specific to wrist stimulation and the response of the third neuron to the tactile stimulation of the thumb or index finger. To quantify the response evoked by each stimulation type, we computed the difference between the minimum and the maximum value of the PSTH in the ±400 ms window. The maps in **Figures 9B–D** illustrate the spatial distribution of the response amplitudes for proximal (elbow, shoulder, **Figure 9B**), proximo-distal (wrist, **Figure 9C**), and distal (hand, fingers, **Figure 9D**) stimulations, respectively. The response amplitude at each electrode is averaged across all SUAs and MUAs discriminated at this electrode location. Red and blue squares indicate strong and weak evoked responses, respectively. As in Section “Mapping the Single Neuron Spiking Activity Across the Cortical Surface,” the three maps were spatially smoothed by averaging the amplitude at each electrode with the amplitudes at all directly adjacent electrodes. **Figure 9** shows a clear distinction between proximal and distal upper limb representations over the cortical surface covered by the electrode array. The neurons at the bottom of the array (medial on the cortical surface) respond much more vigorously to proximal stimulation whereas those in the top left corner (lateral on the cortical surface) are more responsive to distal stimulation. The responses to proximo-distal stimulation are more distributed over the array.

### COMPARISON OF MAPS OBTAINED WITH DIFFERENT SIGNAL TYPES

**FIGURE 10:**
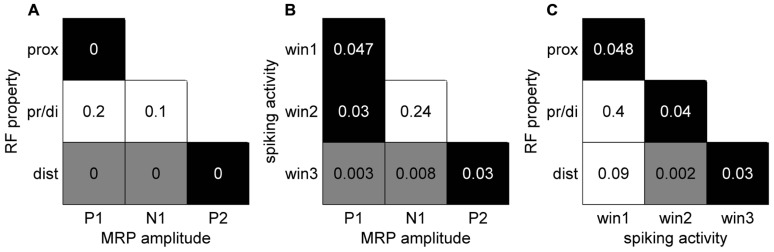
**Significance levels of comparisons between maps of different signal types of neuronal activity**. Black: statistically significant (*p*<0.05) positive map; gray: statistically significant negative map; white: not significant. The numbers correspond to the *p*-value for each combination. **(A)** MRP maps (**Figure 4**) vs RF property maps (**Figure 9**). **(B)** MRP maps vs spiking activity maps (**Figure 8**). **(C)** Spiking activity maps vs RF property maps.

In a final analysis we looked for a relationship between the MRP maps, the RF maps and the maps of peak spiking activity. We used our bootstrap procedure to compare these different maps. In summary (**Figure 10**), a significant match was observed between all combinations of (i) the representation of proximal somatosensory RFs and passive movements around elbow and shoulder (**Figure 9B**), (ii) the amplitude maps of the P1 component of the MRP (**Figure 4A**), and (iii) the map of the percentages of neurons peaking during the corresponding time window (win1 in **Figure 8C**). Furthermore, there is a close match between (i) the maps of the distal somatosensory RFs on hand and fingers (**Figure 9D**), (ii) the representation of the late component (P2) of the MRP (**Figure 4A**), and (iii) the distribution of the percentages of neurons peaking during the same time window (win3 in **Figure 8C**).

## DISCUSSION

We showed that, in motor cortex, the MRPs of the LFP are characterized by complex spatio-temporal properties during the execution of reach-to-grasp movements. Although our data are only from one monkey, our results obtained over more than 7 months of recording (see Data Selection) were highly reproducible, suggesting a general finding. For each individual MRP component, the peak amplitude and its latency with respect to movement onset vary along the cortical surface following a precise structure. We observed that these spatial modulations are related to the firing properties of the single neurons recorded in the same cortical area. In addition, we also showed that the spatio-temporal properties of both the LFP and the spiking activity may be linked to the spatial organization of the somatosensory inputs to motor cortex, as estimated by RF testing.

### COMPLEX SPATIO-TEMPORAL PROPERTIES OF THE MRPS DURING REACH-TO-GRASP MOVEMENTS

It has previously been shown that during reaching movements, LFPs exhibit a large MRP around movement onset containing three to four clearly distinguishable components (Donchin et al., 2001; Roux et al., 2006; Kilavik et al., 2010). In the present study, we show that even five distinct components can be identified during reach-to-grasp movements. We suggest that the striking difference in the MRP structure between reaching and reach-to-grasp movements relates to their difference in complexity. When compared to a reaching movement, reach-to-grasp movements additionally involve a tight coordination between arm and hand movements so that the hand is already preshaped when contacting the object (Jeannerod, 1984). Grasping movements also require a fine control of the contact forces for object manipulation and this control is closely dependent upon the cortical processing of somatosensory inputs from the hand and fingers (Picard and Smith, 1992; Brochier et al., 1999; Salimi et al., 1999). These additional processes activate dedicated cortical circuits projecting onto the hand area of motor cortex (Tokuno and Tanji, 1993; Dum and Strick, 2005) where they directly modulate the activity of layer V neurons during grasp (Tokuno and Nambu, 2000; Kraskov et al., 2011). In the present study, two additional observations support the assumption that the complexity of the MRP reflects the movement-related modulations of motor cortical activity. First, we showed that the spatial distribution of the peak amplitude and its latency differs between the early (P1/N1) and late components (P2/N2/P3) of the MRP (**Figures 4A,B**). This difference suggests that the processes giving rise to the early and late components are, at least in part, spatially segregated. Since the early components are systematically observed in both reaching and reach-to-grasp tasks, they are probably related to unspecific preparatory motor processes (Roux et al., 2006) or the motor control of the reaching part of upper limb movements (Gemba et al., 1981). The later components (P2/N2/P3) are more specific of reach-to-grasp movements and would reflect the activation of grasp-related local networks in M1. Second, we observed that the structure of the MRP was consistently more complex during execution of PG rather than SG trials. In particular, the P2 component could be subdivided in two subcomponents with distinct topographies (**Figures 4C,D**). Previous work in human and non-human primates indicate that PG is characterized by a greater level of complexity and is more demanding in terms of neural control (Ehrsson et al., 2000; Begliomini et al., 2007). Grasping an object between the tip of the thumb and the index finger leads to more instability than grasping an object with a whole hand grip and requires additional sensorimotor control mechanisms (Johansson, 1996). In line with this idea, the subdivision of the P2 component occurs right before the object touch (**Figure 2**) and may indicate the activation of specific processes for the control of a PG.

### SPATIO-TEMPORAL RELATIONSHIP BETWEEN SPIKING ACTIVITY AND MRP COMPONENTS

Previous works suggest that during LFP oscillations, spiking activity increases during the negative peaks of the LFP, indicating that LFP reflects the synchronization of excitatory inputs to the neurons around the electrode tip (Baker et al., 1997; Destexhe et al., 1999; Denker et al., 2011). Following this hypothesis, the motor command originating from layer V in the motor cortex should produce a sustained negativity in the recordings. However, to our knowledge, there is no evidence that the relationship between spike rate and LFP negativity holds for the MRPs (see Discussion in Roux et al., 2006). We observe that the MRP presents a robust alternation of positive and negative peaks throughout movement execution. **Figure 8B** shows that a majority of the recorded neurons are maximally active between 40 and 140 ms after switch release, in close temporal relationship with the P2 component of the MRP (**Figure 4**). Many fewer neurons are showing a peak of activity later than 140 ms after switch release, when the large N2 component of the MRP is observed. Although we did not assess the direct temporal coupling between spike and LFP, our observations suggest a non-systematic relationship between LFPs and firing rate during movement execution. In particular, the comparison of LFP and spiking data shows that the MRP expresses at least five distinct components (see **Figure 2**), whereas neurons tend to present a single peak of spiking activity around movement onset (see **Figure 7**).

### RELATION TO PROXIMAL–DISTAL REPRESENTATIONS

In agreement with earlier studies (Rosén and Asanuma, 1972; Lemon, 1981), we observed that a large proportion of motor cortical neurons were responsive to passive stimulation of the upper limb. RF testing with the 100-electrode Utah array presented two additional advantages. First, the RFs were tested simultaneously on all electrodes, making thus sure that the same mechanical stimuli were used for all neurons. Second, we could directly reconstruct the spatial distribution of the RFs at all electrode locations and compare the RF maps for distal and proximal stimuli. Using this approach, we demonstrated that the proximal and distal parts of the upper limb were preferentially represented toward the medial and lateral sides of the array, respectively. This spatial organization is reminiscent of motor cortical maps obtained by ICMS in which a representation of the hand and fingers close to the central sulcus is surrounded by a representation of the arm toward the arcuate sulcus (Kwan et al., 1978; Park et al., 2001). Although ICMS effects were not tested in the current experiment, the comparison between our RF maps and ICMS maps in earlier studies confirm a close match between the afferent input and motor maps in the motor cortex (Rosén and Asanuma, 1972; Lemon, 1981).

Furthermore, we analyzed how this spatial organization of the motor cortex is modulated during complex reach-to-grasp movements. Our results show a clear shift of neural activity from medial to lateral motor cortex during the course of the movement that is revealed both in the MRP and in single neuron firing rates. This shift of activity occurs between the N1 and P2 components of the MRP and between the corresponding temporal windows for the peak spiking activity. In terms of behavior, these temporal windows correspond to the MT between movement onset and object touch (Jeannerod, 1984). Importantly, the spatio-temporal structure of the MRPs and spiking activities closely match the spatial distribution of the RFs. During the early part of the movement (corresponding to the P1 and N1 components of the MRP), the neural activity predominates in the areas receiving somatosensory input from the arm, whereas during the later parts (corresponding the P2, N2, and P3 components), the activity shifts to the hand-related areas. These observations suggest that the underlying organization of motor cortex in terms of body representation strongly influences the modulation of neuronal activity during movement execution. It is, however, important to stress that these spatial modulations are only relative, so that the lateral motor cortex is not entirely silent when the medial part is active and vice-versa. For instance in the MRP, we observed a clear P1 component on all the electrodes, but this component was substantially larger toward the medial electrodes. This indicates that the whole motor cortex covered by the array is activated during the task and that the global pattern of activity is locally modulated in relation to the functional requirements of the different parts of the task. Such organization would be adapted to enable the tight coupling between proximal and distal upper limb segments during reach-to-grasp movements (Wing et al., 1986; Jakobson and Goodale, 1991; Chieffi and Gentilucci, 1993).

It has been proposed that proximo-distal coupling for upper limb movements may be mediated by traveling waves of LFP beta oscillations across the surface of the motor cortex (data filtered at 10–45 Hz in Rubino et al., 2006; Hatsopoulos et al., 2011; Takahashi et al., 2011). In these studies, oscillations were analyzed both in the delay period preceding the movement and during movement execution itself, a period in which beta oscillations are known to be almost suppressed (Kilavik et al., 2012a,b). An attractive hypothesis would be that the transfer of information during movement execution is also mediated by traveling waves in the low frequency range of the MRP (3–15 Hz). This hypothesis would predict that the latency of each MRP component should vary along a given trajectory across the array, but that the amplitude of the peak at these different latencies should remain constant. We observed instead that the MRP peaks varied in amplitude in direct relationship with the peak latency, i.e., the smaller the peak, the later the latency. This correlation rather suggests that each MRP component derives from a local source and that the peak at a remote electrode from the source occurs later and is of smaller amplitude. However, more detailed analyses will be required to confirm this hypothesis.

Altogether, our results show a clear spatio-temporal structure of the MRP and spiking activities over the motor cortex that relates to the proximo-distal organization of this cortical area. This organization would provide the essential substrate for the control of complex reach-to-grasp movements involving the coordination of multiple segments of the upper limb. However, it is likely that other properties of the recorded area are also contributing to the spatio-temporal representation of the neuronal activity. In particular, since our electrode array was implanted over M1 and PMd (see **Figure 1C**), area-specific activity modulations may also come into play. But this issue cannot be directly addressed, since our data do not allow a clear distinction between these two areas.

## Conflict of Interest Statement

The authors declare that the research was conducted in the absence of any commercial or financial relationships that could be construed as a potential conflict of interest.
